# Synthesising key findings from Indonesia’s first civil society-led forum on primary health care: PHC forum, Jakarta, 13-14 November 2023

**DOI:** 10.1186/s12919-025-00338-0

**Published:** 2025-08-20

**Authors:** Diah Satyani Saminarsih, Clarissa Cita Magdalena, Nadhira Febianisari, Yurdhina Meilissa, Sadika Nuraini Hamid, Muhammad Iqbal Hafizon, Sayyid Muhammad Jundullah, Olivia Herlinda

**Affiliations:** Center for Indonesia’s Strategic Development Initiatives, Jakarta, Indonesia

**Keywords:** Primary health care, Community, Health equity, Civil society

## Abstract

**Background:**

Despite evidence showing the important role of a robust primary health care (PHC) in achieving health equity, many nations still need strong, directional policies and unyielding commitments to strengthening PHC after the Astana Declaration. The “PHC Forum: Towards Health Equity and Resilient Communities” was held in November 2023, in Jakarta, Indonesia, to discuss the challenges and opportunities in actualising the commitments towards a robust PHC system globally.

**Methods:**

This paper presents a thematic analysis of the PHC Forum. The Forum was organised into two days with a total of 783 participants, categorised as policymakers, academics, health workers, civil society leaders, and community members, mostly from the Global South countries. The Forum aimed to democratise stakeholders participation by creating an inclusive platform for all relevant actors, including civil society organisations (CSOs) and community health workers (CHWs). This was intended to generate empirical insights derived from lived experiences. To synthesise key findings from the Forum, expert statements were systematically compiled, categorised, and triangulated with relevant prior studies.

**Results:**

The Forum resulted in the identification of key messages that can be organised into seven themes: (i) health for all vision, reinforcing that investing in a robust PHC system is central to achieving the universal health coverage (UHC); (ii) community participation and empowerment, reiterating community not just as beneficiaries; (iii) importance of CHWs, with a focus on realising decent work; (iv) financing and prioritisation, calling for increased investment towards PHC; (v) innovations, underlining the role of innovative technologies and approaches to improve health equity; (vi) PHC integration, stressing the need for both vertical and horizontal integration of public and private health services; and (vii) climate change, with an emphasis on recognising the increasingly critical role of a robust PHC system in facing climate change.

**Conclusions:**

The key messages that emerged from the Forum emphasise the essential elements for building a robust PHC system, particularly in the Global South. A paradigm shift beyond health to focus on the bigger systems is needed to achieve health for all. The Forum emphasised that communities are the leaders in PHC transformation, driving towards equitable health outcomes.

## Background

Following the Alma Ata Declaration in 1978, primary health care (PHC) has gained global recognition for its essential role in strengthening health systems [1]. This shift towards PHC is critical for addressing ongoing and new health challenges [2]. Research consistently shows that a robust PHC system improves public health, makes health systems more efficient, and reduces health inequities [1–3]. The evidence highlights the significant benefits of investing in and committing to PHC for various public health outcomes.

Regrettably, numerous nations that have grappled with setting strong, directional policies and unyielding commitments for PHC often face extended trajectories to eventual success [3, 4]. The COVID-19 pandemic highlighted our collective failure to establish health systems fortified by robust PHC fundamentals [5]. The pandemic responses gravitated towards an emergency "firefighting" approach, emphasising acute incident management strategies and procedural standards, involving mainly hospitals [5]. In multiple countries, majorly in the Global South, partly due to the long-standing systemic insufficiencies, PHC workers reported closure of routine services, sudden shifting work dynamics and heightened stress, impacting the communities and the health workers [6–8].

Different past experiences, existing capacities, and governance strategies in countries resulted in diverse extents and methods of response to the pandemic [7, 8]. Looking at this trend of different responses, many nations realised the pivotal role of PHC following the aftermath of the pandemic, utilising distinct strategies, culminating in divergent yet uncertain trajectories for health systems' resilience. Open dialogues emphasising meaningful participation are required to ensure that the lessons gained during the pandemic are not lost but shared among nations [8–10].

Acknowledging each nation's strengths and best practices to fortify health system resilience, the Center for Indonesia's Strategic Development Initiatives (CISDI) is committed to creating a space in which global actors can convene and rethink longer-term health goals based on shared learnings following the pandemic. From 13 to 14 November 2023, CISDI ran the “PHC Forum: Towards Health Equity and Resilient Communities” in Indonesia. The forum restarted the dialogues on PHC, from defining community resilience to financing, involving Global South and North actors, prompting future knowledge co-creation and collaboration.

While the ongoing global discussions continue to emphasise the transformative approach to PHC systems, including through high-level forums, there remains a critical gap in inclusive consultative processes that ensure equitable participation of civil society, including marginalised communities. The PHC Forum established an intentionally egalitarian platform in which all participants, from grassroots community health workers (CHWs) to policymakers were invited to speak on equal footing. This inclusive design positioned the Forum as a unique and elevated space that facilitated dialogue across multiple levels of society and warrants proper documentation. In light of the limited documentation of such community-driven, Global South-focused events on PHC, this paper seeks to synthesise the PHC Forum’s key messages and conclusions, thereby contributing to and enriching the global discourse on PHC through a more grounded and participatory lens.

## Methods

This paper employs a thematic analysis to derive key messages from the PHC Forum that was organised into two days, with a plenary session and five competence forums on the first day and more plenary sessions on the second. A total of 783 participants from 11 countries attended the forum, with more than 90 per cent representing Global South countries. The participants varied from leaders to implementers from multiple sectors with varied affiliations, such as government ministries, donor partners, United Nations agencies, non-government organisations, private enterprises, academia, youth activist groups and the media.

Participants were engaged in the plenary sessions and competence forums through live question-and-answer sessions or active discussions. We define competence forums as sessions that are more interactive and collaborative, such as talk shows and workshops. In the talk show format, a moderated panel of speakers shared their experiences and perspectives in a conversational setting, followed by audience interaction. In the workshop format, participants were more actively engaged in smaller breakout groups facilitated by facilitators from the host organisations. Three of the authors acted as rapporteurs, working alongside ten additional non-author rapporteurs to take the notes during the Forum. Notes were written live during the sessions using standardised note-taking templates, consisting of the speaker's name and verbatim notes as the main components, followed by key messages and conclusions, which are completed after the note-taking.

The data extraction and analysis process was conducted in several stages. Three authors first reviewed the Forum notes and ensured they were complete and accurate by listening to the audio recordings. Key insights and important comments were then extracted through a deductive thematic approach. Key insights were categorised into themes that represented the core issues discussed in the Forum, which are health for all, resilient communities and PHC, importance of community health workers (CHWs), financing and prioritisation within PHC, innovations in PHC to improve health equity, PHC integration, and PHC and climate change. The authors subsequently triangulated the findings with prior studies to generate a cohesive narrative, situating them within the broader context of the research. Three authors independently check the recording notes, categorisation and final results and discussion narratives, this alongside cross-referencing with current evidence mitigated some bias, although residual bias may remain and is acknowledged as a limitation of this synthesis.

While several forums on PHC have been organised at both national and global levels, the decision to use the Forum as a data source due to its unique characteristic as a civil society-led forum in Indonesia, with particular attention to the Global South. Its inclusive and participatory approach, designed to engage actors from grassroots to policymaker levels, provides insights relevant to ongoing discussions on PHC systems strengthening. In this context, the Forum contributes to broader post-Astana dialogues on advancing more community-driven approaches to PHC.

Ethics approval was not applicable considering this report did not investigate personal data. All speakers quoted in the manuscript were informed that their names and quotations would appear in this report, and gave their written consent to publication.

## Results

The authors identify several key messages, grounded in both evidence and experiences shared by a diverse group of stakeholders across sectors who attended the Forum. These key messages can be grouped into the following seven themes: health for all, resilient communities and PHC, importance of CHWs, financing and prioritisation within PHC, innovations in PHC to improve health equity, PHC integration, and PHC and climate change. Collectively, they reflect foundational elements of a robust PHC system essential for accelerating health equity achievement.

### Health for all

Prof. Kent Buse used the term “Healthy Societies Vision” to situate health for all in a broader context, namely in which society makes efforts to keep people healthy and free of the need of health care services through attention to the structural drivers of ill health, including through effective delivery of health in all policies [11]. The vision refers to societies where everyone may enjoy universal and indivisible human rights, including the right to health and to a healthy environment, which is more likely to be achieved through meaningful, representative, accountable, transparent, and fair governance mechanisms [11].

Progress will be made by focusing on fixing systems, not people. For example, by fixing food systems, transport systems, housing systems, social protection systems, countries can move towards realising the vision. In addition, Prof. Kent Buse argued that there needs to be a radical redistribution of the 5Ps (power, privilege, possibility, prosperity, and participation) to realise the vision.

As the cornerstone of health systems, PHC is positioned not only to ensure access to essential health services but also to address the social determinants of health. Strengthening PHC systems is therefore indispensable to strengthening the right to health and achieving resilient communities, as envisioned in the “Healthy Societies Vision.”

### Resilient communities and PHC

Community participation and empowerment are key components of the PHC system, reiterated throughout the PHC Forum. Communities can be active participants, not just as beneficiaries but also as providers of care and planners of the PHC system [5]. In turn, PHC delivered by the communities can be an important source of strength during crises or conflicts, delivering care when resources become scarce [12].

To elevate the discussion on the role of communities in the PHC system, the committee invited diverse panellists to discuss how PHC systems across countries should involve communities from design to the evaluation process. This includes how to co-create innovations and increase communities’ roles in the social accountability of PHC governance. Through our panel discussions, we aim to emphasise a paradigm shift in which collaboration between government and communities as non-governmental actors is no longer an option but a fundamental approach to provisioning PHC for all.

Abdul Malik, then Minister of State for Gender, Family, and Social Services, Republic of Maldives, re-emphasised that communities are not homogenous. Engaging diverse communities, from indigenous to non-indigenous, and considering the rich different contexts and knowledge are crucial to achieving a more acceptable and responsive PHC [13]. Efforts are required to avoid a one-size-fits-all approach in PHC delivery. Indigenous communities deeply understand health as holistic, predating modern definitions of well-being, and acknowledging their longstanding knowledge of what constitutes health is required as a first step towards providing responsive care [13, 14]. Traditional healers in these diverse communities must be embraced and integrated into the broader health system.

In the Maldives, the community, traditional health workers have been integrated at the PHC level with medical professionals. It has contributed to eradicating malaria and polio. Maldives has also made efforts to shift from modernised healthcare focusing on curative services to strengthening PHC and preventive measures by engaging with communities [15]. Led by the Ministry of Health, the World Health Organisation, and the Ministry of Gender, Family and Social Services, an initiative creating inclusive policy dialogues through island councils and community groups emphasises community participation to realign the PHC system with community needs.

Community participation is pivotal in ensuring the PHC can deliver care as needed, especially during crises. For example, when the Indigenous communities in Alaska commended health care, the COVID-19 case-fatality ratios were lower than in the USA as a whole, insinuating the care systems were more resilient [16]. It was discussed at the Forum that during the recent conflicts, communities have had access to health care through health workers in PHC, which was also driven by the communities. In the conflict, as people face significant obstacles to accessing basic health services, local initiatives and civil disobedience movements led by health professionals became lifelines to provide health services for communities. Robust mechanisms that involve active and meaningful community participation in PHC systems thus ensuring the system answers to diverse needs, may lead to resilient PHCs and, hence, communities.

### Importance of community health workers

The significance of CHWs in PHC should no longer be a subject of doubt, and recognising their vital role has emerged as a central concern throughout the Forum. In the early 1970s to 1980s, the first CHW programs in South and Southeast Asia were initiated, particularly in the Philippines, Pakistan, Indonesia, India and Nepal [17, 18]. CHWs were tasked with delivering community health services, mainly in rural areas. Up until now, they play vital roles in providing health services to the most vulnerable in many low- and middle-income countries (LMICs) [19].

A competence forum session on driving salaried, skilled, supervised, and supplied CHWsilluminated the global variations in CHWs’ status and conditions. CHWs are not always compensated and valued based on their roles [20, 21]. The core notion that their roles should be driven by volunteerism is still prevalent among stakeholders. It is not separated from the gendered assumption that women primarily provide unpaid care willingly, especially since most CHWs are women [20, 21]. Insecure working conditions also partially inhibit CHWs’ roles in bridging the health care gap in communities. CHWs working without policies that ensure structured roles, incentives, and safety measures are most likely to quit or perform suboptimally [19, 20].

The Forum re-emphasised that decent working conditions, wages, and safety measures for all PHC workers are necessary for effective health service delivery, and CHWs should not be an exception [20, 22, 23]. There is a need to overcome hurdles such as obtaining recognition of CHWs as workers from the community to policymaker level, thereby enabling decent working conditions with gender transformative principles and addressing the gendered notion of care work [20, 23].

### Financing and prioritisation within PHC

Adequate, efficient, and equitable financing systems that offer good value for money while fostering innovation are central to strengthening PHC, thereby guaranteeing accessibility and affordability in healthcare [24]. With political support and modest additional investments, PHC can become the catalyst for health systems resilient to future challenges, including pandemics, climate change, and the burden of infectious and non-communicable diseases [2, 25]. A cost–benefit analysis in Kenya predicted that every $1 invested in PHC interventions saves up to $16 in spending on conditions like stunting, NCDs, anaemia, TB, and malaria [26].

WHO has suggested that governments allocate at least 1% more of their gross domestic product (GDP) to PHC to achieve UHC [27]. To increase the financing pool, stakeholders were suggested to find innovative ways to increase tax space that could be used for investments in PHC. Innovative financing based on tax or non-tax should also be deployed to increase fiscal space for cost-effective PHC investments. Innovative mechanisms such as sin tax, debt swaps, development and social impact bonds can address resource mobilisation challenges [28–30].

However, a competence forum session on innovation pathways and prioritisation in PHC emphasised that governments require evidence and realistic assessments to assist them in prioritising which PHC interventions should be funded aside from just increasing the financial supply. Evidence is required so that efficient allocation of the health budget for PHC can be made. Shifting allocation from funding curative to preventive interventions, is also deemed necessary to reduce health spending in the future [24, 31].

Determining the intervention packages with the strongest evidence of outcomes and cost-effectiveness for strategic purchasing is strongly recommended. It can persuade key stakeholders, including the Minister of Finance and international donors, to allocate resources towards PHC [25, 32]. It was expressed that a recent study had identified three key investment areas for donors to strengthen PHC in achieving UHC: community empowerment, particularly for women, new models of people-centred PHC, and investing in the next generation of CHWs, all with integrating digital or new technologies [25].

Health technology assessment (HTA) can be a powerful tool for assessing other key priorities for funding. It can help determine context-relevant, cost-effective key interventions that have yet to be included in national health benefits packages [32]. The competence forum session on innovation pathways and prioritisation in PHC highlighted a case from Pakistan, whereby the HTA employs the multistakeholder's Evidence-Informed Deliberative Process (EDP) to design cost-effective benefit packages that cater to diverse community perspectives. With evidence-backed prioritisation, sustained investment and effective allocation will result in well-equipped PHC facilities, appropriate technology utilisation, and better access provision for the community.

### Innovations in PHC to improve health equity

To improve health equity, PHC can capitalise on innovations that assist equitable service delivery, from planning to evaluation. Disruptive innovations can lead to enhanced health products and services catering to the diverse needs of multiple population groups [33]. However, to ensure innovations are expanded and integrated into the current system, an enabling ecosystem must be created that promotes innovations [34, 35].

One panellist suggested that enabling an ecosystem involves seamless, transparent, and robust adoption and procurement regulations that enable private companies to develop and market health innovations for the public benefit following careful reviews by the public sector. An example is how the Digital Transformation Office of the Ministry of Health Indonesia integrated data and services into the national digital health platform (Satu Sehat) [36]. The platform attempts to integrate data from more than ten thousand clinics, three thousand hospitals, laboratories, and pharmacies. Specific to the PHC, My Health Indonesia (abbreviated ASIK) application collects and integrates data from Indonesian CHWs in the field.

Enabling an ecosystem for innovation also means the openness of each stakeholder to collaboration in creating and maintaining health innovations. For example, in the Forum, PATH showed a human-centred-design innovation co-developed with multiple stakeholders from Kenya called the NCD Navigator. It is a web platform that showcases Kenya NCD data, monitors strategy implementation, and guides resource allocation (accessible on https://ncd-icc.or.ke/dashboard). It has been continuously redeveloped, considering the public and stakeholders' inputs [37]. Other technologies shown in the Forum that have grown due to partnerships include point-of-care testing or diagnostics tools at the PHC level that have expanded in India, for example Smart Scope® cervical cancer screening tool and the Niramai Thermalytix breast cancer screening tool [38].

Innovations do not always involve digital technology. All actors may contextualise different kinds of innovations based on community needs. A notable form of non-digital innovation is the social referral mechanism, which enables communities to provide supplementary food to families with malnourished and undernourished children under five. This initiative is locally funded, by and from the community, and implemented through the coordinated efforts of CHWs, village officials, and health workers [39].

However, in the Forum it was also advised that despite the rapid advancement of technology, ethics and professionalism should remain paramount. The issue of data privacy, for example, needs to be carefully considered during innovation design or implementation [40].

### PHC integration

Vertical integration, which connects services across different levels of health facilities or between various tiers of government, and horizontal integration, which fosters collaboration between public and private providers at the same level, are essential for enhancing the accessibility, affordability, and acceptability of PHC [41, 42]. Vertical and horizontal integration enhances continuity of care, seamless transition, coordination, and consistent, comprehensive, standardised care. The goal is for people to access quality care seamlessly and efficiently, regardless of location and various means [41, 42]. Integration is also required to adopt the innovations described in the previous section into the care pathways.

In various countries in Asia and Africa, people access the private facilities for their health care [43]. Factors driving people towards private care include convenience, comfort, privacy, and better quality services, all of which are especially important for marginalised groups such as sexual and gender minorities and people with disabilities who may feel unwelcome or stigmatised by public providers [44, 45].

Private health care can be unaffordable without government stewardship and regulations to ensure public–private collaboration that benefits communities. Effective policies are required to increase access to plural providers [5, 24, 33]. A competence forum session on the role of design thinking, partnerships, and innovations in transforming PHC systems emphasised the need to integrate private health services into national health insurance schemes, while also highlighting the importance of partnerships. A civil society organisation representative pointed out, among others, the good practice of partnerships with pharmacists and social enterprise clinics for early detection of diseases such as TB, HIV, diabetes, and hypertension.

However, concrete incentivising mechanisms, regulations, and monitoring tools are required to sustain public–private partnerships (PPP) [24, 46, 47]. First, the governments can allocate structural, organisational, or financial incentives for private sectors, such as pay-for-performance or bundled payments for care episodes, to incentivise private sectors to provide quality care [24, 48]. However, emphasis should be put on incentivising practices that benefit society, such as preventive services and establishing private practices in high-burden areas [24, 48].

Ultimately, an ideal PHC system involves well-regulated, high-quality services, allowing individuals to choose between the public and private sectors [41, 42, 47]. Emphasising the importance of inclusivity, perceived quality of care, and convenience, there is a crucial need for cross-learning and applying best practices between the private and public sectors [41, 42, 47].

### PHC and climate change

A particular focus in the Forum was given to the ongoing threats from climate change. Climate change will worsen the existing burden of climate-sensitive diseases in the region, including malnutrition, vector-borne diseases, and maternal, newborn, and child deaths [49]. The COVID-19 pandemic has further exposed and multiplied these health risks, especially in the face of high levels of inequality, inadequate social protection, and heightened ecosystem damage and biodiversity loss [50]. A competence forum session, dedicated to discussing this important topic, highlighted that climate change disruptions disproportionately affect coastal, indigenous, remote, and forest communities.

Increased health risks and burdens mean overstretched health systems. Typhoon Rai in 2021, which impacted parts of the Philippines, including coastal and remote islands like Cebu and Siargao serves as a notable example.Jit Sohal, former Regional Climate Manager at the Health Care without Harm explained that Typhoon Rai disrupted healthcare services in affected areas, leading to about 400 deaths with some due to acute gastroenteritis and inadequate maternal care. The incident highlights communities' vulnerability and health facilities' inadequacy in facing climate-related health challenges. Therefore, investing in strengthening communities and frontline providers in PHC is crucial to face future shocks [51, 52].

To ensure sustainable health services and protect community health in the future, PHC systems should be adaptable to climate change and contribute to mitigating further damage [53]. In providing responsive services and goods, PHC is the anchor of a health system with strength, security, and resilience to absorb shocks and prevent crises [50, 53]. It was noted in the session that a holistic approach encompassing political will, economics, behaviour, and psychology, with the utilisation of technology, is required to address threats of climate change to the health system. In reaching remote communities, whose health and safety will be affected by climate-induced disasters, governments must ensure policies and technology are inclusive to prevent disparities in access to health services and climate-health mitigation or adaptation programs.

## Conclusions

The PHC Forum 2023 in Indonesia brought together multidisciplinary stakeholders to exchange ideas, suggestions, real-world evidence, and insights on advancing the PHC system, particularly in LMICs, with the objective of achieving health equity and resilient communities. The Forum highlighted that in transforming PHC systems globally, it is critical to include underrepresented communities in the conversations, foster integrated service delivery and cross-sectoral collaboration, ensure robust accountability and transparency at all levels, and promote equity in access and outcomes. These principles collectively reflect the values of inclusivity, diversity, integrity, accountability, equity, and transparency.

Furthermore, achieving an equitable PHC system calls for a comprehensive approach, which necessitates strong political commitment and significant investment. It requires coordinated policies and actions across multiple sectors, such as education, infrastructure, environment, and social welfare, among others. This holistic strategy is key to advancing UHC in the face of challenges like the climate crisis and future pandemics. Evidence-based, targeted interventions that are measurable and adequately financed are vital, and innovative financing mechanisms can drive substantial progress in PHC. Creating environments that incentivise innovation is also important. Technology and digitalisation, in particular by providing predictive data, can enhance PHC by accurately assessing stakeholders' diverse needs, allowing for tailored interventions and resource allocation.

The Forum provided evidence that communities are at the forefront in driving PHC system transformation and will remain essential to achieving equitable health for all, not merely as beneficiaries, but as active partners. The documented experiences and lessons learned serve as a strong foundation for future discussions on building a more resilient PHC system both within the region and globally.

## Data Availability

Not applicable.
